# CSF Dynamics for Shunt Prognostication and Revision in Normal Pressure Hydrocephalus

**DOI:** 10.3390/jcm10081711

**Published:** 2021-04-15

**Authors:** Afroditi Despina Lalou, Marek Czosnyka, Michal M. Placek, Peter Smielewski, Eva Nabbanja, Zofia Czosnyka

**Affiliations:** 1 Brain Physics Laboratory, Division of Neurosurgery, Department of Clinical Neurosciences, University of Cambridge, Cambridge CB2 0QQ, UK; mc141@medschl.cam.ac.uk (M.C.); mp963@cam.ac.uk (M.M.P.); ps10011@cam.ac.uk (P.S.); e.nabbanja@addenbrookes.nhs.uk (E.N.); zc200@medschl.cam.ac.uk (Z.C.); 2 Institute of Electronic Systems, Faculty of Electronics and Information Sciences, Warsaw University of Technology, 00-661 Warsaw, Poland

**Keywords:** hydrocephalus, normal pressure hydrocephalus, CSF infusion tests, CSF dynamics, intracranial pressure, cerebral blood flow

## Abstract

Background: Despite the quantitative information derived from testing of the CSF circulation, there is still no consensus on what the best approach could be in defining criteria for shunting and predicting response to CSF diversion in normal pressure hydrocephalus (NPH). Objective: We aimed to review the lessons learned from assessment of CSF dynamics in our center and summarize our findings to date. We have focused on reporting the objective perspective of CSF dynamics testing, without further inferences to individual patient management. Discussion: No single parameter from the CSF infusion study has so far been able to serve as an unquestionable outcome predictor. Resistance to CSF outflow (Rout) is an important biological marker of CSF circulation. It should not, however, be used as a single predictor for improvement after shunting. Testing of CSF dynamics provides information on hydrodynamic properties of the cerebrospinal compartment: the system which is being modified by a shunt. Our experience of nearly 30 years of studying CSF dynamics in patients requiring shunting and/or shunt revision, combined with all the recent progress made in producing evidence on the clinical utility of CSF dynamics, has led to reconsidering the relationship between CSF circulation testing and clinical improvement. Conclusions: Despite many open questions and limitations, testing of CSF dynamics provides unique perspectives for the clinician. We have found value in understanding shunt function and potentially shunt response through shunt testing in vivo. In the absence of infusion tests, further methods that provide a clear description of the pre and post-shunting CSF circulation, and potentially cerebral blood flow, should be developed and adapted to the bed-space.

## 1. Background

### 1.1. Rationale and Significance of the Review

It has been over 50 years now since infusion tests were introduced directly into bedside practice, as a method of assessment of the CSF dynamics and circulation systems.

Thirty years elapsed since, we started performing them in Cambridge hydrocephalus clinic day cases and neurosurgical inpatients [1,2,3,4]. In those decades, conviction about the overall benefit of their use has fluctuated from very optimistic, to cautiously optimistic, and perhaps difficult to adapt due to ambiguous benefit for some neuroscience centers [5,6,7,8,9,10,11]. Despite the objective and quantitative information derived from testing of the CSF circulation, there is still no conclusion on what the best approach could be in defining, selecting for shunting, and predicting response to shunting for those patients.

### 1.2. Hydrocephalus: Clinical and Radiological Perspective

Hydrocephalus is currently perceived as either the life threatening, acute ventriculomegaly with increased intracranial pressure, or the more chronic form of “normal pressure” hydrocephalus, that could potentially be reversed by augmentation of patient’s CSF outflow by a shunt. An accurate, careful process of selecting those patients for shunting [12,13,14,15,16,17,18] is crucial. However, the heterogeneity of the disorder is well known, and the limitations of studying all patients that may be contained within a grey zone between those two extremes and extending into various subcategories are challenging to address. When considering further into the disease’s pathophysiology and management, we can understand that hydrocephalus is far more complex than a disorder of CSF circulation and CSF dynamics. The most adopted clinical approach is along the symptoms-imaging axis.

In most cases of hydrocephalus patients, we would most likely be trying to interpret a CT or rarely an MRI with obviously dilated ventricles, with or without signs of periventricular lucency. Radiological indications of raised pressure, cerebral atrophy, and ex-vacuo dilatation [19,20,21,22] are not always reliable. These findings, supplemented by the clinical presentation and clinical examination, constitute the semi-quantitative, and generally subjective, clinical assessment of hydrocephalus. They will also guide further decision making and management until the patient is either discharged from any neurosurgical pathway, shunted, and sustainably improved or failed to improve despite shunting.

### 1.3. Importance of Investigating NPH: Where a CSF Flows; Role of Reversal Flow & CSF Dynamics

Based on the current definition and clinical axioms of this decision process, the reports of improvements are between 40–80% of shunted cases [23,24,25,26,27,28,29,30]. We are still left wondering, after many decades of using clinical symptoms plus brain imaging, since the first description of NPH, why 20–60% patients will not respond to the shunt treatment. And most importantly, we can conclude that by limiting ourselves to using only the core clinical-imaging axis, we are most likely to miss an adequate information to support our decisions. Similarly, when these patients present to the follow-up clinics lacking any improvement post-surgery, with persistent symptoms and generally unchanged neuroimaging, we do not possess objective, effective tools to understand why the shunt operation has failed to reverse the patient’s symptoms. Furthermore, we should not forget that the decision to implant a shunt is not the only source of ambiguity and risk. The principal challenge of implanting a shunt would extend over life-time period of follow-up. That involves dealing with other complications not related to the immediate post-operative time period, including further decisions to proceed to a shunt adjustments’ revision. This includes weighing the risks and benefits of each investigations and course of action for the years to come in both responders and especially in non-responders [31,32,33,34,35,36,37].

The role of assessment of CSF dynamics is particularly important from the perspective of the treatment of hydrocephalus patients. Insertion of a shunt dramatically alters the CSF circulation and hydrodynamics properties of the cranio-spinal system. Therefore, the knowledge about the system that we are going to modify and manage in the long term, appears to be vital. In many, if not all cases, apart from checking CSF dynamics, it should involve CBF studies [3,38,39,40,41].

In this review, we aimed to demonstrate the lessons learned from assessment of CSF dynamics in our center and summarize our findings to date. We do not aim to make any clinical recommendations as these findings are mainly centered on the science behind CSF circulation testing, as well as retrospective comparison with clinical practice and outcome. We will simply give an overview of the CSF dynamics perspective, a very focused and specific perspective compared to the overall individual clinical approach of patients in modern, personalized medicine. 

## 2. Infusion Test Methodology and Parameters

### Principles of Bedside CSF Infusion Tests

CSF infusion tests, the procedure, data recording, analysis, and interpretation have been exhaustively detailed in numerous publications and do not fall within the scope of this review [2,4,5,9,39,42,43,44,45,46,47,48,49,50,51,52,53,54,55,56,57]. A comprehensive summary of the meaning and clinical significance of each parameter will be briefly described in the relevant sections. Safety of the procedure is of utmost importance in the clinical settings. Direct access to the cerebrospinal fluid is needed. A lumbar puncture (LP) is usually performed when no pre-implanted device exists, and patients are carefully evaluated for possible contraindications. The test can also be performed via an extra ventricular drain (EVD) or an ommaya reservoir. Some patients with ommaya reservoirs might have previously undergone endoscopic third ventriculostomy (ETV), whereby an infusion test is performed in order to assess the effectiveness or the procedure and/or the necessity of proceeding into shunting. We will not focus on assessing ETV success and outcomes. In shunted patients (or in those with reservoirs in situ), small diameter butterfly needles (25G) are used to access the CSF spaces through the available reservoir. We have validated the safety of the above procedures both through recording absence of any related adverse events and particularly demonstrating very low infection rate on several studies (<1%, averaging significantly lower from a standard LP in a neurosciences ward) [39]. Routine microbiological and biochemical testing is therefore no longer performed, unless otherwise clinically requested. A safety threshold of ICP of 40 mmHg is used; if exceeded, infusion is stopped immediately, and CSF can be drained if required.

**Figure 1:**
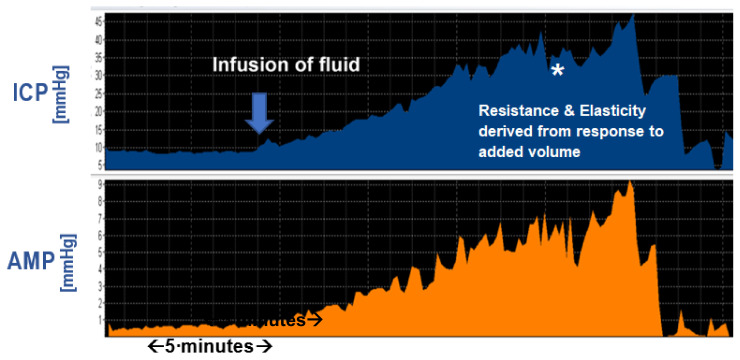
Infusion study recording. Baseline recording of 10–15 min, followed by infusion (starting from arrow) of fluid (Hartmann’s/Normal Saline) with constant rate (1.5 mL/min) until pressure reaches plateau. Resistance to CSF outflow (Rout) is calculated from the difference of the plateau and baseline pressure, divided by the infusion rate. Elasticity (“stiffness”) of the brain, is calculated from the pressure-volume curve produced during infusion. Each step of the grid represents 1 min in time, as marked on the x-axis. ICP: Intracranial Pressure. Amp: Fundamental harmonic of ICP Pulse Amplitude.

Infusion test offers several advantages and alternatives compared to ICP monitoring (Figure 1). Besides their short duration (average 45 min for infusion tests compared to an average of 2-day hospital stays that involve a general anesthetic and more complications for ICP monitoring), they allow for calculation of resistance to CSF outflow (Rout) and elasticity. Several shunt problems can also be better investigated and diagnosed with infusion test [39,43,57,58,59]. Less commonly, we have described the opportunity to flush shunts during infusion tests and clear any obstructions usually associated with the intraventricular catheter [57].

With the advancement of imaging technology, as well as ICP monitoring and shunt technology, infusion tests can easily be combined with different methods. This can be performed in order to assess additional parameters of the cerebral mantle, white matter, and cerebrovascular reactivity, as well as retrieve complementary information required to fully assess patients (e.g., flow assessment devices or telemetry devices) [60,61,62,63,64,65].

## 3. CSF Dynamics: Interpretation of Findings Versus Relationship to Outcome after Shunting

### 3.1. Steady-State ICP (Baseline)

One of the mainstays of our decades of scientific data is that ICP is not a number, but a continuous, complex variable determined by many factors [12,66,67,68,69,70,71,72,73], which we will discuss below. Methodologically, it would be erroneous to expect any of the disturbances in those components to be reflected in a singular, isolated, ICP measurement. First and foremost, we know physiologically that there is a close interaction between CBF and ICP, not only mediated through changes in cerebral perfusion pressure (CPP). As such, disturbances in ICP will arise not only from issues in CSF formation, circulation, and absorption, but there is also an important vascular component [1,74,75,76,77,78]. Depending on the disease and the aetiology of hydrocephalus, this vascular component could contribute a high percentage to the rise in ICP observed intermittently [79,80,81,82,83,84,85,86,87,88,89,90].

**Figure 2:**
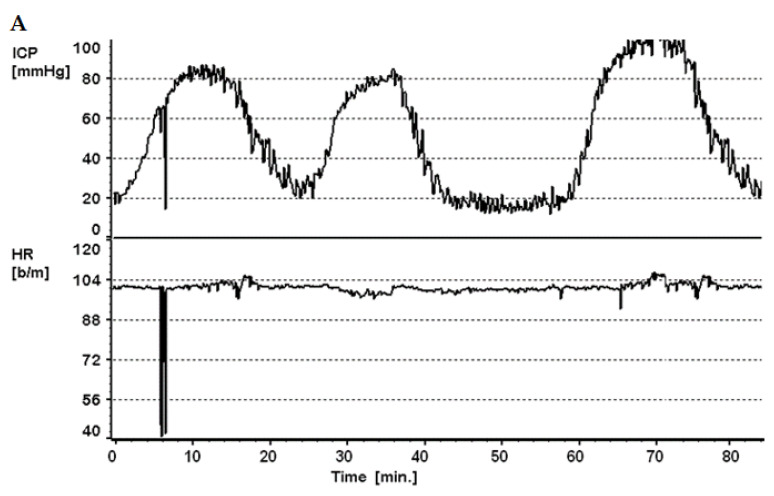

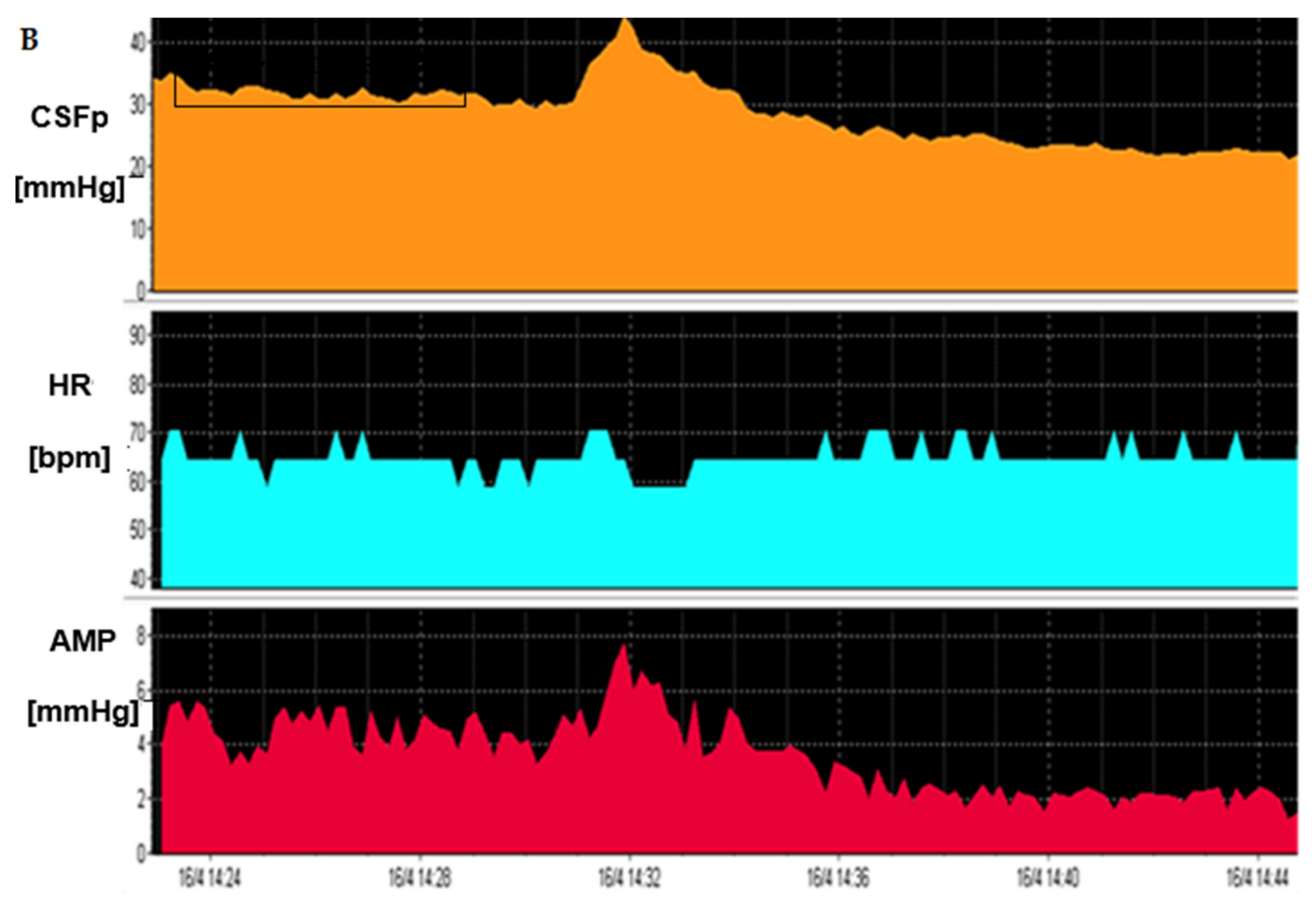
Variability of Intracranial pressure. (**A**): ICP recording (prolonged baseline, no infusion) of a patient with communicating hydrocephalus after SAH. Note regular fluctuation of ICP level from relatively “normal” range of 15–18 mmHg to abnormal waves of >60 mmHg in intervals of 15–20 min, most probably plateau waves of ICP. (**B**): change of ICP reading again from an initial level of 32 mmHg to 22 mmHg over a 10–20 min period in a paediatric patient. Both examples illustrate that ‘ICP is more than the number, and its value may change from normal baseline to high values in a matter of minutes. HR: Heart rate. CSFp: CSF pressure (when ICP not measured with an intraparenchymal bolt).

We have previously illustrated this phenomenon with many examples of long-term ICP monitoring and infusion tests. Figure 2 shows an example of significant variations in ICP revealed by long term ICP monitoring, rather than snapshot manometry. These are examples of patients with suspected CSF disorders, which indeed clinically could not be diagnosed even with repeat manometries until appropriate methodology was utilized to guide diagnosis and subsequent management.

### 3.2. Resistance to CSF Outflow: Calculation, Normal Ranges and Thresholds

**Figure 3:**
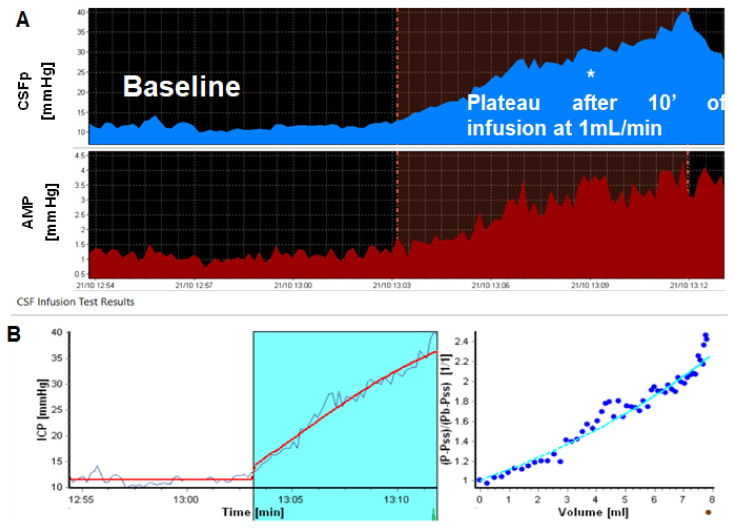
Resistance to CSF outflow (Rout): calculation based on the static formula (ICPplateau—ICP baseline)/Infusion rate (**A**—note that ICPplateau is not always stable, as indicated by *) and dynamic method of non-linear curve fitting (both rise in ICP during the test and derived pressure-volume curve) to a theoretical model built in to ICM + (**B**). ICP/CSFp: Intracranial Pressure/Cerebrospinal fluid pressure. AMP: fundamental amplitude of ICP. On lower panel y axis, the formal (P-Pss)/(Pb-Pss) represents measured pressure (P) during infusion, pressure of the sagittal sinus (Pss), and baseline pressure (Pb).

Figure 3 illustrates the circulatory component of CSF, Resistance to CSF outflow (Rout), calculated as the difference between ICP plateau (ICPp) and ICP baseline (ICPb), divided by the infusion rate (If): Rout = (ICPp − ICPb)/If [5,9,91,92,93,94]. A “normal” CSF infusion test result would be a combination of baseline pressure around 10 mmHg and Rout < 11 mmHg*(min/mL) [9,10,12,14,51,95,96]. “Normal thresholds” are still considered a subject of debate, without any to date widespread scientific and clinical endorsement of validated thresholds for steady-state ICP or Rout [11,12,26,97]. There have been several studies on baseline ICP value ranges in physiological and NPH patients using the baseline average from lumbar infusion tests, confirming an average ICPb of 10–13 mmHg [average value we have detected in subjects with Rout < 13 mmHg*(min/mL)] [9,98,99].

The typical pattern of NPH CSF dynamics consists of normal baseline ICP and a raised Rout. Early scientific experiments in physiological individuals defined the normal range of Rout from 6–10 mmHg*(min/mL) [9,51,96,98,100]. Despite the evidence dating back many decades, the mathematics and methodology used to calculate Rout were almost identical to the ones adapted in our current methodology and ICM+ software. It is worthwhile to note, however, that the distribution of Rout values reported from Ekstedt et al., involved ages 20–80 years, with the majority of patients’ age being in the range of 50–65 and the questions of age variability of Rout have been under investigation since. Repeat studies examined the Rout distribution in healthy elderly populations and reported an increase of the upper limit of normal of Rout up to 18–20 mmHg*(min/mL) [14,93,101,102,103,104].

### 3.3. Resistance to CSF Outflow versus Outcome after Shunting

Studying and validating Rout and other CSF dynamic parameters in physiological individuals naturally provides cleaner data and easier interpretation. Determining a clinically useful threshold, however, has proven a lot more complex and the reasons behind this are multiple and might fall beyond the scope of this review. Based on the physiological data, the hydrocephalus practice in Cambridge has generally been to utilize the threshold of 13 mmHg*(min/mL) in combination with the corresponding clinical and radiological findings of NPH in order to support decision-making for offering shunting to patients. This threshold was based on late 20th century evidence of very optimistic prognostic values of Rout > 12. Twenty-first century evidence has conversely brought to light larger ranges of Rout among shunt responders and non-responders. A summary of the available evidence on Rout is shown in Table 1.

**Figure 4:**
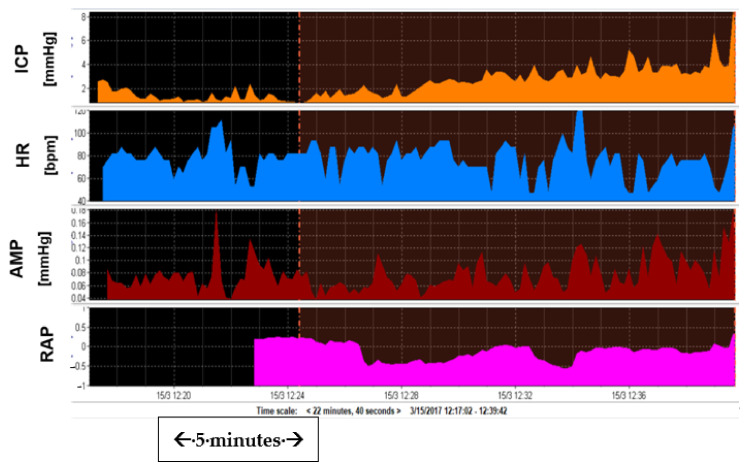
Example of CSF dynamics in cerebral atrophy. Picture of CSF dynamics identical to the decompressed patients: Low Rout (ICP increased from a baseline of 1.3 mmHg to 3.6 mmHg) with very low AMP at baseline (0.08), barely reacting to infusion (AMP plateau 0.09 mmHg). RAP (running correlation coefficient between changes in 10 sec averaged ICP and AMP over 5 min period) at baseline is low (<0.6), never exceeding 0.6 despite infusion of >25 mL.

On a different note, it might be possible to establish a lower threshold for Rout that could indicate significant atrophy, and therefore no response to shunting, or in other cases CSF leak. Figure 4 shows an example of slow response and minimal rise of ICP during infusion, indicating possible cerebral atrophy (increased space in the brain and therefore minimal response of pressure to volume addition) [3,71,76,107]. A separate entity, not discussed in this review and mentioned here only for completion, is pseudotumour cerebri syndrome (PTCS). PTCS is a good example demonstrating the importance of the vascular component of ICP, as it is considered mainly a disorder of venous blood outflow with Rout and CSF formation possibly contributing minimally to its clinical presentation and management [86,108,109,110,111,112,113].

**Figure 5:**
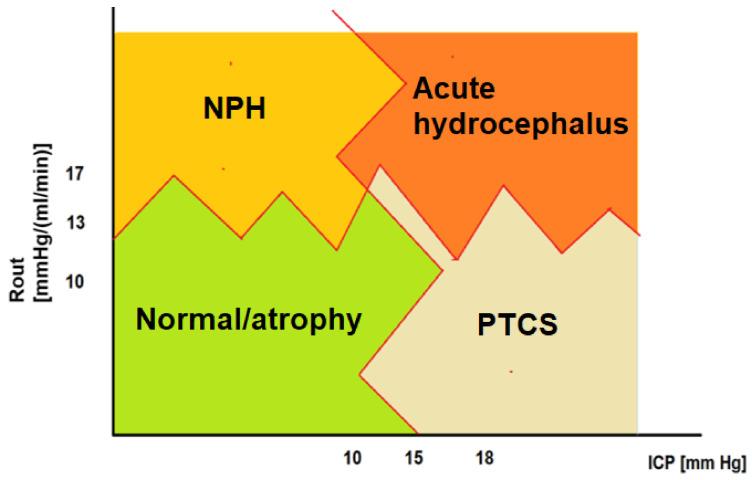
Classification of CSF disorders based on ICP baseline and Resistance to CSF outflow. Rout along the y-axis is plotted against ICP baseline. Using Rout thresholds and ICP thresholds, we have generated four quadrants, separated from each other via irregular borders to reflect the grey areas yet to be elucidated. Normal patients, and in extremes, atrophic brains tend to have ICP < 15 mmHg and Rout < 13 (even lower in atrophy or intracranial hypotension). NPH should show ICP < 15 and increased Rout above 13–17 mmHg*(min/mL). Acute hydrocephalus has both high ICP and high Rout. For completion, PTCS (or idiopathic intracranial hypertension) is shown as a disorder with high baseline ICP but normal or low Rout. (Figure modified from M. Czosnyka, 30 Lectures on Brain Physics, https://www.neurosurg.cam.ac.uk/research-groups/brain-physics-lab/brain-physics-lectures-2020/?fbclid=IwAR0A6Yh-NqAheMO7GHi2HHD-Bug5mgSyhK7nWM1uG729wbt4a6XNqOzFZQ4; accessed on 21 March 2021).

If ICP alone provides us with minimal to no information about the pressure equilibrium in the brain, ICP combined with Rout allows for a rudimentary 2-D classification of CSF disorders. Figure 5 depicts this preliminary classification based strictly on ICP and Rout, bearing in mind that clinical validation and clinical outcomes linked to this classification are variable; there are grey zones (presented here as overlapping, saw-patterns between quadrants) still requiring investigations and perhaps further CSF parameters, which are discussed below and will be required to complete this initial puzzle.

### 3.4. Amplitude and Amplitude-Pressure Relationship

Pulse amplitude can be calculated as the peak-to-peak amplitude of the ICP waveform, i.e., the difference between the systolic and diastolic peak of the wave or as the fundamental amplitude of the first harmonic of the ICP pulse waveform, as calculated in ICM+ [4,114,115]. During a continuous pressure recording, both calculations would yield similar results. Its physiological meaning has been described in multitude references before, and overall, reflects the pulsations of the vascular bed. Focusing on its clinical significance, AMP has shown some relationship with outcome, however, not strong enough in order to be utilized clinically and interpret bedside test. Raised AMP at baseline has been sporadically associated with higher likelihood of improvement after shunting [116,117,118,119,120]. No such relationship has been validated by randomised trials.

**Figure 6:**
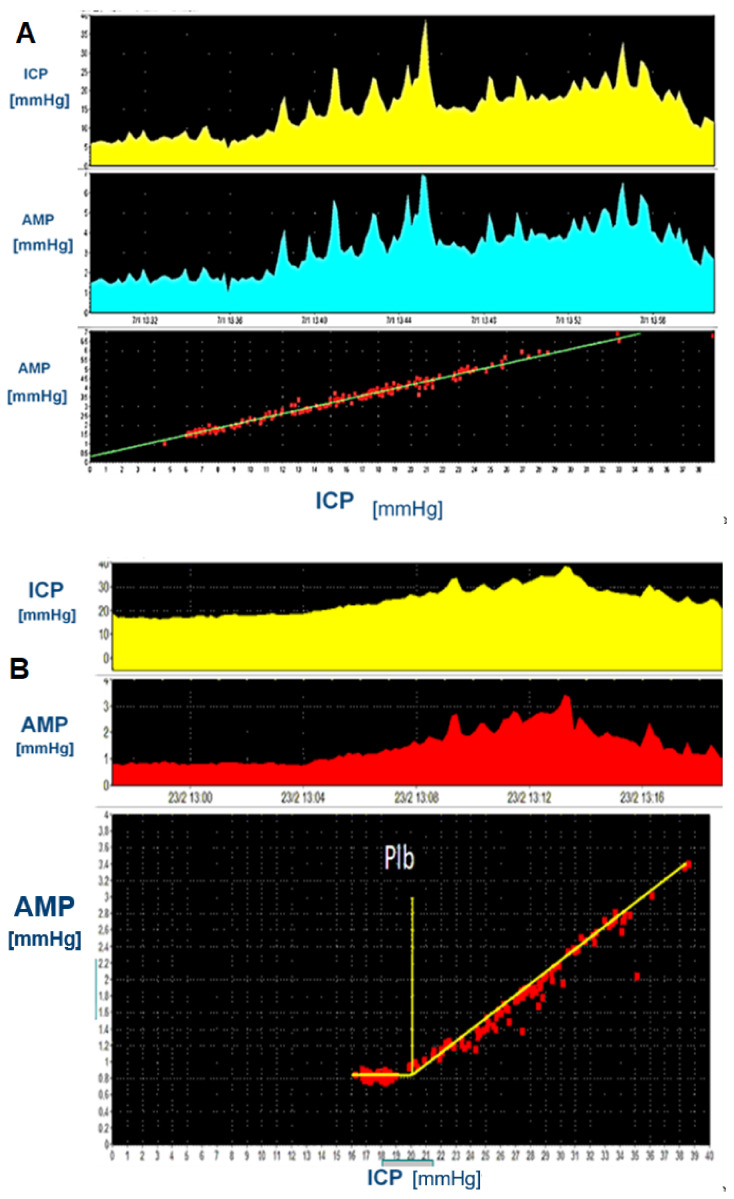
Amplitude-pressure line during infusion tests in Normal Pressure Hydrocephalus (**A**): direct linear relationship between ICP and AMP both at baseline and during infusion. This relationship indicates a possible “tighter” equilibrium of cerebral fluids, with volume addition inducing significantly increased ICP and ICP waves. (**B**): Presence of a lower breakpoint in the AMP-P regression (Plb) after which the previously shown linear relationship begins. Physiologically this could be interpreted similarly as a compliant brain with space for pressure–volume compensation.

During infusion, both in physiological individuals and patients with NPH, AMP rises in close linear relationship to ICP. When plotting this linear relationship during the entirety of the infusion test, the slope of the amplitude-pressure (AMP-P) line can be calculated through simple linear regression. The AMP-P relationship may already be linear from baseline, or show a lower breakpoint, above which a strong linear relationship begins and below which the line become horizontal (Figure 6). An increased slope, representing steeper response to volume addition and therefore a “stiffer” brain, has also been associated with improvement after shunting [48,99,105]. As such, the AMP-P slope directly correlates with elasticity, i.e., the steeper the AMP-P line, the “stiffer” (less compliant) the brain (see Section 3.5 about elasticity).

### 3.5. RAP and Elasticity

Similar to the AMP-P slope, RAP is derived from the correlation of AMP and ICP and calculated as their time-moving correlation coefficient. Lack of a direct linear relationship (RAP closer to 0) overall reflects a state of adequate compensatory reserve in the cerebrospinal compartment, as is expected in healthy subjects, but also in cases of atrophy. As the compensatory reserve becomes depleted, RAP values are closer to 1. Depleted ccompensatory reserve at baseline (i.e. before the start of infusion) has in its turn been associated with higher rates of improvement after shunting. However, RAP calculation during infusion tests can be technically challenging, as it requires at least 5 min windows of clean data, and it is not infrequent for patients to move and generate artefacts during such a period [59,78,107,117,121].

Elasticity has been studied in NPH to a lesser degree compared to all the other parameters, as it plays an uncertain role in NPH pathophysiology. Elasticity has shown no superiority to the remaining pressure–volume compensation parameters in predicting outcome after CSF diversion, with potentially the exception of ETV outcome in patients with aqueductal stenosis [122,123,124,125], where increased elasticity may predict positive response to ventriculostomy. There is no correlation between Elasticity and RAP at the baseline.

### 3.6. Slow Waves of Intracranial Pressure

**Figure 7:**
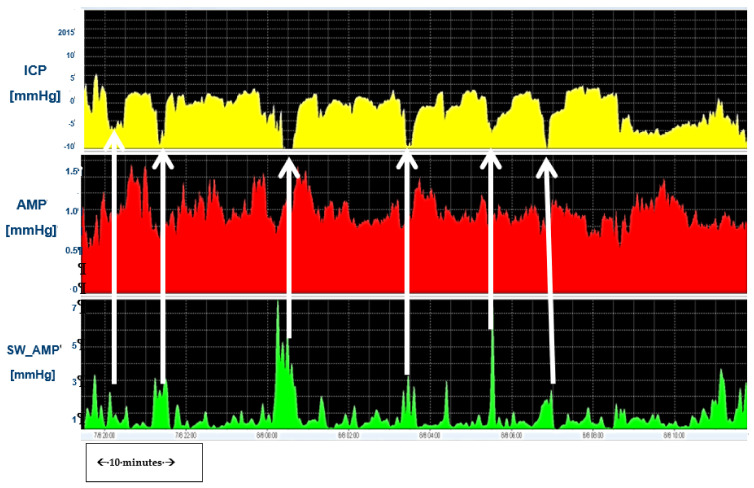
Illustration of CSF over-drainage related to strong slow wave activity. Frequent ICP slow waves (b-waves) as shown in this case (green), result in respective frequent opening of the shunt and drainage of CSF. Subsequent abrupt dips in ICP (yellow) down to a level of −10 mmHg corresponding to the opening and drainage of the shunt valve. A constantly open shunt valve can subsequently cause significant over-drainage Red illustrates changes in pulse amplitude of ICP (AMP). SW_AMP: slow wave amplitude.

Slow waves of ICP are oscillations correlated with the cerebral blood volume vasocycling and can be automatically detected by frequency analysis either during CSF infusion tests or from overnight ICP monitoring [126,127,128]. It has been difficult to clearly distinguish physiological from pathological b-waves, as well as demonstrate their potential prognostic value [3,118,129,130,131]. Conflicting data exist to date as to the relationship of increased magnitude or frequency of occurrence of slow waves, with clinical improvement after shunting in NPH. That remains a question to be addressed [25,66,83,132,133,134,135,136,137,138,139]. To the best of our knowledge, no specific numerical thresholds have been accepted for the frequency and magnitude of slow wave that described NPH or other CSF disorders. In Cambridge study we accept threshold 1.5 mm Hg of effective averaged amplitude of slow waves.We have shown though that b-waves are significantly attenuated by general anesthesia [126]. Interestingly, a way in which strong b-waves could influence outcome after shunting is that they could lead to shunt over-drainage as a result of frequent opening of the valve and constant intense CSF drainage (Figure 7).

### 3.7. Cerebral Blood Flow and Autoregulation in Hydrocephalus

There is currently very weak evidence about any diagnostic and/or predictive value of CBF and autoregulation in NPH. However, there is a need to address the CSF-CBF crosstalk. Examples from traumatic brain injury patients with intracranial hypertension attribute rises in ICP to vascular rather than CSF factors up to 70% of the time [76,140,141,142,143,144,145,146,147]. It is unknown how frequently and how much vascular factors and particularly CBF influence CSF dynamics in NPH and vice versa.

What we can observe through the use of infusion tests and analysis of ICP components, in combination with other methods, are the following:

#### 3.7.1. Slow Waves and Vasomotor Reactivity

**Figure 8:**
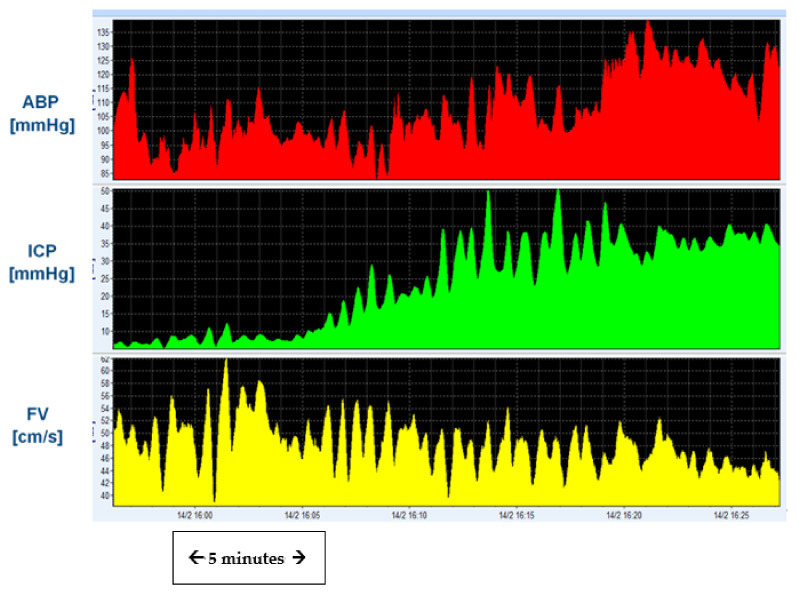
Slow waves of ICP, arterial blood pressure and cerebral blood flow velocity. Simultaneous recording of mean arterial blood pressure (ABP), ICP, and transcranial doppler derived flow velocity (FV) recording through the middle cerebral artery. Note the similarities in frequency and synchronicity in slow waves of FV and ICP. Cerebral autoregulation indices can be calculated from both ICP and ABP, as well as FV and CPP (ABP–ICP).

Slow waves, as observed in ICP recordings during infusion tests, are sometimes referred to as vasogenic waves due to the dominant, but not proven, explanation of their origin [83,126,136,148]. Through recordings with transcranial doppler during infusion tests, they appear to be in close synchrony with the slow waves of arterial blood pressure (ABP) and cerebral blood flow velocity (Figure 8). These slow waves of intracranial pressure, blood pressure, and cerebral blood flow velocity carry autoregulatory information and provide a method of assessment of cerebral autoregulation concomitantly to CSF pressure and CSF circulation [132,134,135,139,148,149,150,151,152,153,154,155,156].

The coefficient Mx is calculated as time moving window correlation between the slow waves of cerebral flow velocity and cerebral perfusion pressure. Another reactivity index, PRx, could be assessed during infusion tests using the slow waves of ICP and preferably non-invasive ABP (invasive ABP monitoring is of course ideal, however, it will have its own limitations and is not practical to perform routinely for our day cases). The more positive Mx and PRx are, the more “passive” the cerebral vascular & pressure system, therefore, autoregulation is impaired and ABP or even ICP changes would passively result in impaired CBF [7,60,90,142,143,157,158,159,160].

#### 3.7.2. Resistance to CSF Outflow and Autoregulation

**Figure 9:**
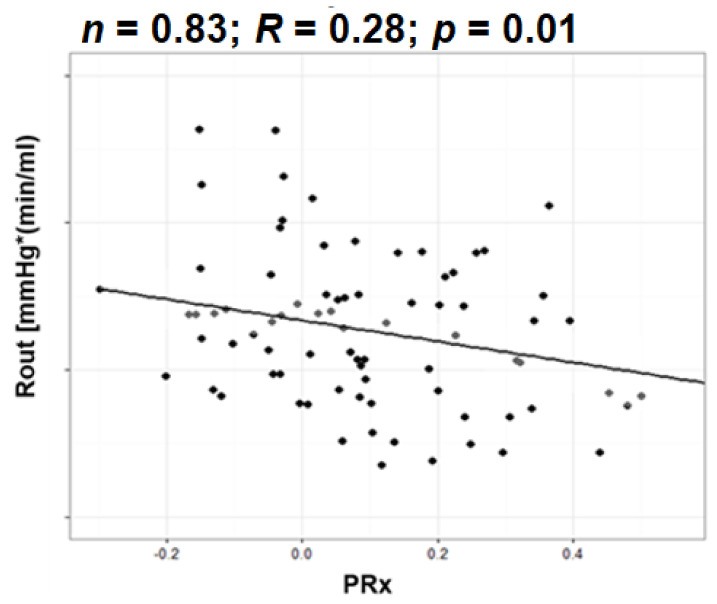
Inverse relationship between Rout and PRx. Data derived from lumbar infusion studies, who were selected for CSF diversion. The 83 patients included had been selected for shunting based on general clinical workup and discussion of risks versus benefits. Higher resistance and negative PRx indicate deranged CSF circulation with overall preserved global autoregulation, potentially highlighting a pathology amenable to shunting.

In patients with clinical/radiological symptoms of NPH, the two indices described in a previous point (Mx and PRx) are known to negatively correlate with Rout. With high resistance patients tending towards preserved autoregulation, compared to those with normal and lower normal Rout, who tend towards positive values (Figure 9) [7,60,161]. Interpreting those results clinically once again proves difficult without any further investigations, including appropriate MRI imaging for periventricular white matter lesions and assessing the same relationship, as well as the reversal of the CSF and autoregulatory/CBF disturbances post-operatively [14,63,84,97,162,163,164,165,166,167,168,169,170,171]. We could say that those fortunate enough to suffer from a purely circulatory issue that has not affected their autoregulation could have the best chance at improving after shunting. They have a reversible CSF disorder and have been spared the devastating effects of deranged CBF regulation and subsequent exposure to long periods of ischaemia and/or hyperaemia. On the other hand, those with normal Rout and impaired autoregulation might have already reached a state of irreversible damage and/or may suffer from advanced cerebrovascular disease that should not be managed as a CSF disorder.

**Figure 10:**
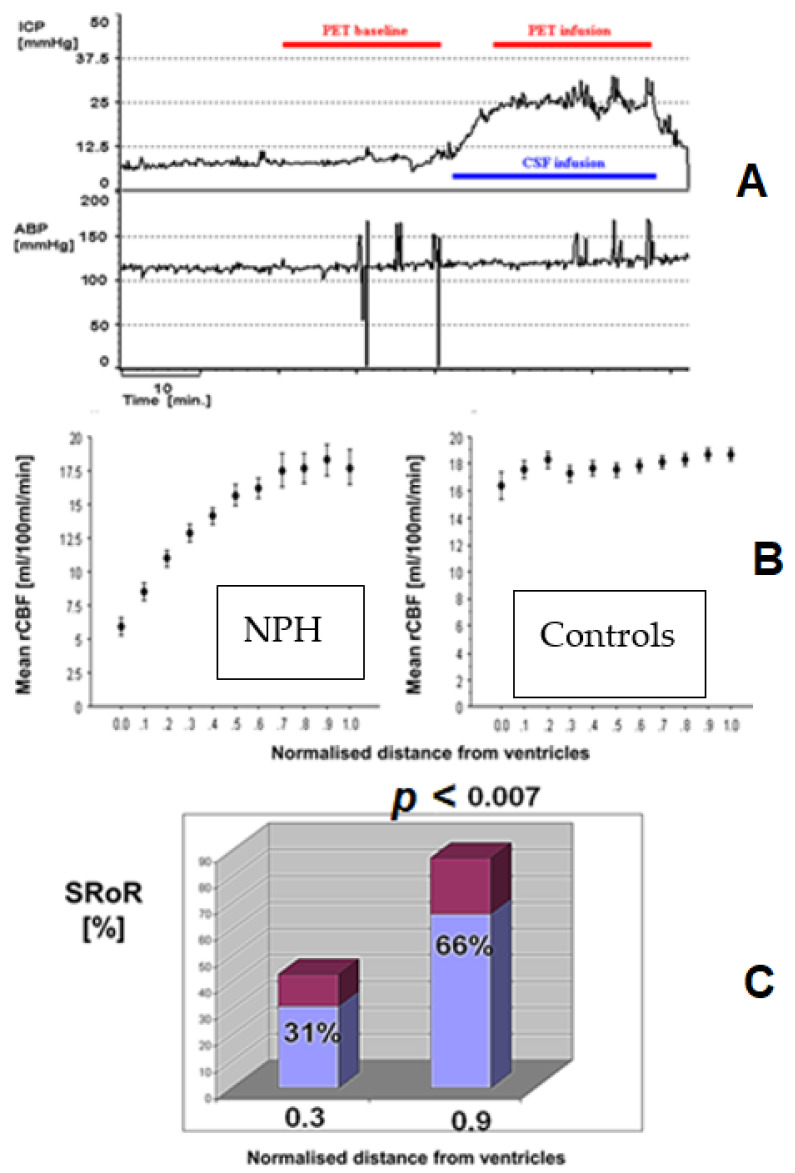
Regional cerebral blood flow and autoregulation in hydrocephalus: relationship with distance from ventricles. (**A**): Demonstration of methodology from our previous investigation of cerebral blood flow (CBF) using PET H2O whilst performing a simultaneous infusion test to monitor ICP. (**B**): PET study at baseline for recordings of CBF (before infusion test). The mean regional CBF (rCBF) in NPH (**left**) was significantly reduced in proximity to the ventricles compared to normal, aged-matched controls. (**C**): ICP monitoring allowed for calculation of the static rate of autoregulation (SRoR), which demonstrated that autoregulation of CBF is similarly significantly impaired in NPH at the regions in close relationship with the ventricles. SRoR is derived from the change in cerebral perfusion (Mean Arterial Blood pressure–ICP) and CBF during infusion. SRoR 100% indicates ideal autoregulation and 0% absent autoregulation. ABP: Mean arterial blood pressure.

A more precise, albeit limited in clinical practicality, the right method to address relationship between impaired autoregulation, reduced baseline CBF, and the resulting cerebral and clinical impairments, is neuroimaging. Using PET/MRI combined with infusion tests, we have managed to explore the relationship between Rout, cerebral perfusion pressure, CBF, and autoregulation in NPH. What we showed in healthy, age-matched volunteers was that CBF and autoregulation appear constant and unchanged globally, without regional variations. Global CBF and autoregulation appeared reduced in NPH subjects; however, this was influenced by significant regional impairment of those values in the white matter most proximal to the ventricles (Figure 10) [63,64,65]. Furthermore, by using infusion tests for ICP monitoring parallel to PET- CBF measurement, we had been able to measure the cerebral blood flow autoregulation. For that purpose, the static rate of autoregulation (SRoR) was calculated. SRoR is derived from the change in cerebral perfusion (Mean Arterial Blood pressure–ICP) and CBF during infusion. SRoR 100% indicates ideal autoregulation and 0% absent autoregulation. Unlike CBF measurement, calculation of autoregulation provides us with further information on possible underlying mechanisms and pathophysiology of CBF/CBF-ICP derangement in CSF disorders.

### 3.8. Shunt Testing In Vivo

The events that will follow a shunt implantation are the most significant ones, as the procedure itself is nowadays adequately safe and aimed at minimizing mortality and morbidity. What indeed could eventually guide the decision and planning of shunting NPH patients, would be the ability to deal with the sequelae of managing an intracranial device and its many complications and malfunctions [15,30,34,37,172,173,174,175,176,177,178,179,180,181,182,183,184]. Yet the process of assessing patients after shunts implantation remains limited to standard assessment of their clinical symptoms, and imaging that could not reveal any useful information, as it is known that ventricles will not increase and decrease in those chronic patients as they would in patients with acute hydrocephalus [21,22,43,185,186,187,188,189]. Knowledge of the functioning status of an implanted shunt is crucial in determining the outcome and further management of the patients. Without objective testing, it is a blind and highly speculative territory. The first step towards gaining more insight and information into the CSF circulation post shunting, is understanding the basics of how an implanted shunt changes the CSF circulation.

Our methodology of in vivo shunt assessment is based on experience in studying CSF dynamics and data from the UK shunt Laboratory. The method uses an infusion test and appropriate interpretation of the results.

**Figure 11:**
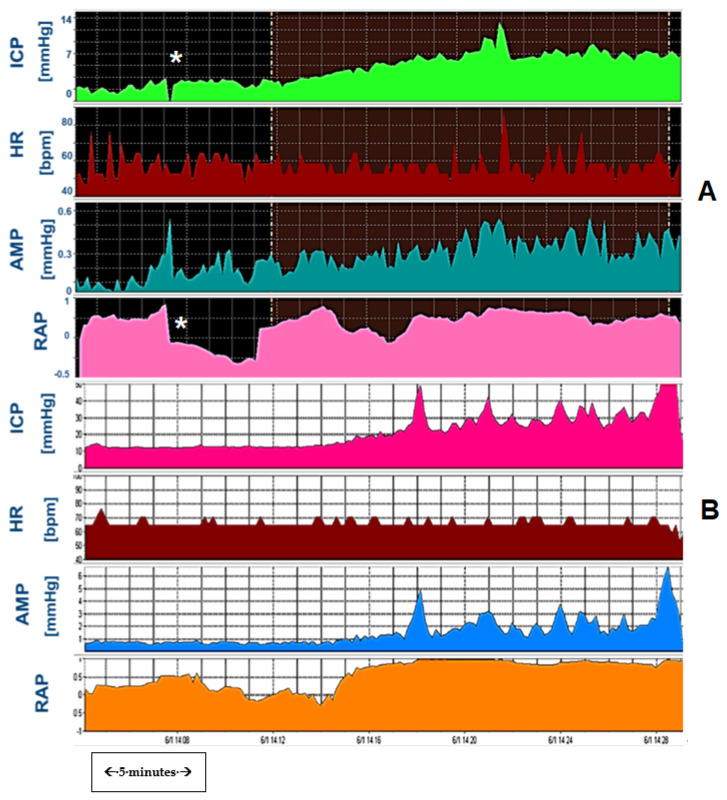
Shunt infusion study results. (**A**): Normal study: Normal ICP at baseline and plateau ICP below critical pressure and normal compensatory reserve (RAP around <0.6). Asterisk: movement artefact, causing artificial dips and spikes in the recorded parameters. (**B**): distal obstruction, During infusion, ICP exceeded the shunt critical pressure [inherent property of each manufactured shunt type, calculated by the formula Critical ICP = Rout*Infusion rate + Shunt operating pressure + Intra-abdominal pressure (5 mmHg)]. The rise in pressure was significantly above the critical pressure, rising to levels >40 mmHg, despite initial ICP within the normal range. Note that pulse amplitude of ICP is well detected, indicating ventricular drain to be patent.

Based on the data from the shunt testing in-vitro, we can assign specific attributes to the implanted shunt system, which appears to be crucial for functioning and consequently an assessment of the implanted shunt. These are: opening pressure, below which no drainage take place; shunt’s hydrodynamic resistance (usually low, 3–5 mmHg*(min/mL)); and a critical pressure, the pressure value which an adequately working shunt should not allow to be exceeded during infusion test [41,190,191,192,193,194,195,196]. The frequency and epidemiology of shunt failure is well known [31,197,198]. Insidious shunt malfunction, that cannot be confirmed clinically or radiologically, is expected to be far more common in complex NPH patients with comorbidities. A shunt infusion test can reliably confirm and exclude shunt malfunction by utilizing the existing knowledge on hydrodynamic properties characteristic of each commercial device that has been reported from testing in the shunt laboratory and adapted for patients in vivo [40,59,178,192]. In the same way that a steady-state ICP measurement in patients before shunt implantation is methodologically inadequate, all dynamic parameters (and not just a random ICP snapshot) need to also be tested when interrogating an implanted shunt. In NPH, the baseline pressure of course will not show any elevation, therefore, assessment of the shunt response to additional extortion during infusion test is effective in evaluation of implanted system. The most important information is contained in how the device is responding to added volume; what are the resistance and plateau pressure? Figure 11 shows examples of a distally obstructed catheter, as visualised during infusion due to significantly exceeded critical shunt pressure and verified intraoperatively.

An important point to bear in mind is that any recorded ICP signal should be pulsatile; if no appropriate ICP pulsation (specifically AMP-pulse amplitude) is detected, then whatever pressure is being recorded does not correspond to ICP. Therefore, if in the pressure signal detected from the shunt prechamber, the pulse amplitude is not detected, this is a prerequisite for conclusion about proximal obstruction.

The number recorded could be a random pressure in the isolated shunt pre-chamber not consistent with real patient’s ICP. Empirical assessments of shunt patency through “pumping” on the pre-chamber repeatedly or attempting to aspirate both to assess proximal patency and to aspirate large volumes of fluid are not only misleading but potentially dangerous; derived dangers are due to the fact that these interventions generate uncontrollable negative pressure in the ventricular system. We have previously demonstrated, in a lab environment, how pumping can generate severe intracranial hypotension, even down to −230 mmHg [199].

Shunt testing in vivo, in its entirety, is a subject on its own and involves diagnosing many other complex issues that could occur. We have described them in numerous occasions, including underdrainage, over-drainage and postural over-drainage, and partially obstructed ventricular catheters. The conclusion that the chosen shunt did not possess hydrodynamic parameters suited to the individual’s CSF dynamics and drainage requirements [3,38,71,106,200] is not uncommon.

In summary, before deciding on a patient’s outcome, it is essential to gather as much objective evidence as possible that the shunt system functions adequately. Unless non-responders are tested further for such possibility, outcome measurement is rendered null and void. If in a research setting we were able to test more shunt responders (as there is currently no clinical indication that would make this acceptable), insights into the CSF circulation post-shunting in the two categories (responders vs non-responders) could help us understand more about the reasons of shunt failure in NPH. Finally, securing adequate CSF drainage, in balance with avoiding the consequences of over-drainage in the elderly, constitutes a separate issue that requires consideration. It is likely that patients with low baseline ICP, low resistance to CSF outflow, and no overnight spikes in ICP above values that will allow the shunt to open and drain CSF may still not be receiving adequate drainage, or any drainage at all, and as such, any inferences related to their outcomes are also invalid.

Even extended lumbar drainage, which ensures a controlled amount of CSF drainage, could subsequently not correlate to shunt response in such a scenario. Interestingly, extended lumbar drainage, which provides controlled drainage over a prolonged period of time (3–5 days), could provide more reliable information in relationship to outcome, as the confounding factor of variable drainage is accounted for. However, 3–5 days of drainage by themselves are often inadequate for assessing response in NPH, as it has been shown that improvement in many cases could be delayed and could take several weeks or months [12,14,23,80,201,202,203,204,205].

Considering all of the above, concluding who requires a revision and who does not, using CSF dynamics appears an easier question to answer than when to implant a shunt in the first place. Consequently, we have been able to address this question in a clinical as well as a theoretical context and have shown the feasibility and safety of shunt infusion tests both in paediatric and in adult patients with non-acute hydrocephalus [39,40,43,57]. Additionally, we have reported and discussed the benefits conveyed to patients and to healthcare systems through objective shunt testing, averaging at least 2 quality years away from hospitals and neurosurgical tables and around 1 million GBP annually respectively [39].

**Figure 12:**
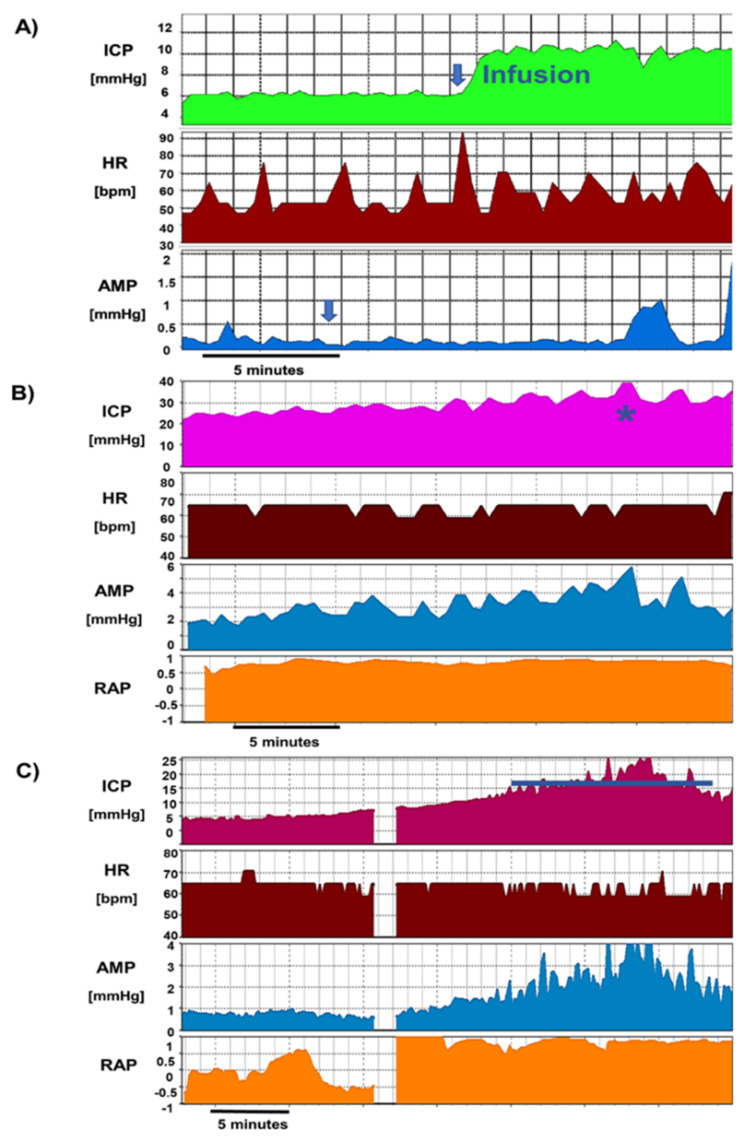
Malfunctioning shunts. (**A**) Proximal obstruction: no pulsatile pressure detected, steep jump in pressure after start of infusion, and no signs of pulsatility during infusion. (**B**) Distal obstruction: clearly raised, pulsatile baseline pressure. (**C**) Underdrainage despite patent distal end: ICP plateau slightly exceeding critical pressure, Rout mildly raised. Distinction between underdrainage and distal obstruction is difficult. Slightly raised Rout and plateau pressure from expected values usually indicate against distal obstruction. Increased intra-abdominal pressure could be considered, or closer monitoring of the patient due to risk of future obstruction. The data gap in this panel is due to artefact removal (artefact had been generated due to patient movement).

To summarise, from a CSF dynamics perspective, some examples who should undergo shunt revision surgery are shown in Figure 12:

- Pulsatile ICP, within the expected limits for the shunt operating pressure, normal resistance, and critical pressure (inherent property of the shunt) not exceeded during infusion: no revision, the shunt is patent and draining well.
- Non-pulsatile “pressure” at baseline, steep jump in pressure after start of infusion, and no signs of pulse waveform recovery during infusion: proximal obstruction. If distal catheter is patent (seen by stabilization of pressure after infusion), it can proceed to revise only the proximal catheter.
- Clearly raised, pulsatile baseline pressure: distal obstruction (or significantly increased pressure on the drainage site, e.g., in raised intra-abdominal pressure). Ventricular catheter patent can revise only the distal end (or lower valve setting if as above increased intra-abdominal pressure is suspected).
- Normal opening pressure, with significant rise in ICP during infusion to values clearly exceeds shunt critical pressure; raised Rout: distal obstruction (Figure 12).
- ICPp slightly exceeding critical pressure; Rout mildly raised: mild underdrainage, consider adjusting the setting and monitor, which may require revision later.

### 3.9. Objective Testing of Cerebrospinal Fluid Dynamics: The New Era

The issue of post-operative shunt functioning brings us back to the problematic relationship between CSF dynamic parameters and clinical outcome.

We have summarised below the main lessons learned from our cumulative work of both research as well as clinical implementation of CSF dynamics. We are highlighting the fundamental principles of balancing scientific principles and clinical practice by avoiding caveats of using either approaches in isolation (only objective testing or only limited clinical/semi-quantitative testing).

#### 3.9.1. Resistance to Cerebrospinal Fluid Outflow Thresholds

Unfortunately, as shown recently by the randomized European iNPH trial, as well as single-centre analysis, CSF dynamic picture and particularly Rout are far from perfect outcome determinants [11,105]. Rout in thresholds >13 and even >18, remains an important objective marker of pathological CSF circulation [10,38,92,100,206]. We should not however utilize Rout as a single number cut-off for determining patients’ outcomes, as we have known throughout the span of these 3–5 decades of CSF dynamics studies that variable estimation with numerical value has limitations. From measurement error due to all kinds of reasons, incorrectly leveling the body during LP, CSF leak around the lumbar needle, to age-dependence of Rout, and the presence of different comorbidities (e.g., cerebrovascular disease). There are multiple factors that could influence the value of Rout and its measurements. More parameters should be considered, including AMP, RAP, slow waves, and cerebral autoregulation, in order to improve our perception as well as tailor our investigations to the individual, rather than base them on singular values and blind analysis of bulk cohorts without any scope or end-game.

However, it should be reiterated that an evaluation of hydrodynamics of the cerebrospinal compartment is helpful in patients management, particularly from the perspective of treatment: with a shunt, by which we interfere with the CSF circulation system.

#### 3.9.2. Cerebrospinal Fluid Dynamics versus Outcome

In order to investigate the value of those added parameters in shunt prognostication, we have recently performed an analysis of 369 patients from our center that were shunted as a result of their CSF dynamics and full clinical investigations [39]. We were able to confirm that age-adjustment of Rout improves its predictive value and that no singular parameter derived from the infusion test could predict outcome accurately. Rout, however, remains the strongest predictor. Finally, the strongest predictive accuracy from combining multiple infusion test parameters, including Rout, AMP-P slope, and ICPb, did not exceed 70%.

These relatively disheartening numbers could lead us to abandoning any effort of testing CSF circulation, as 70% predictive power is not superior to shunting everyone based on the presence of ventriculomegaly and clinical symptoms. It would not be prudent though to rush to such conclusions before considering everything we have discussed in this review. Firstly, we could conclude that CSF dynamics are misleading and of no clinical use. This would entail us to dismiss all data and evidence summarised in this paper and preclude us from taking a step back and thinking that the limitations we face could be in the CSF dynamics, or equally in the way we currently diagnose, follow-up, and define shunt response. Considering all the missing information on how much a shunt in situ drains in NPH and how occult NPH shunt malfunction/obstruction or inadequate drainage may be, shunt response should be carefully reconsidered and redefined. Moreover, given our opening statement on the complexity of NPH that includes a strong vascular component and interaction with the CBF, a CSF circulation and pressure-volume compensation descriptor could more successfully be combined with an autoregulation/ pressure reactivity descriptor, as initiated by the existing studies on CBF and autoregulation in NPH.

To close the circle of the CSF dynamics—improvement after shunting continuum, we bring you back to the argument that objective testing of CSF dynamics should be followed by objective definition and post-shunting description of shunt response. No response to shunting is rarely followed-up to such completion, as there are currently limited tools available (besides CSF infusion tests).

**Figure 13:**
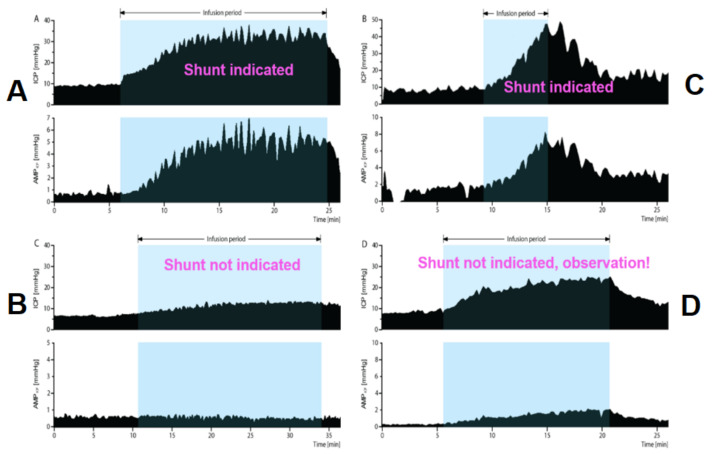
Who requires shunt from a CSF dynamics perspective? (**A**): increased resistance to CSF outflow, frequent and prominent slow waves of ICP (b-waves) are considered indicators of significant disturbance of CSF dynamics and such patterns have not been described in physiological individuals. (**B**): very low Resistance to CSF outflow, dampened and unresponsive amplitude of ICP (AMP), indicating that shunting will most likely not alter the CSF circulation impactfully and that the amount of atrophy and/or lack of cerebrovascular bed reactivity signify irreversible changes. (**C**): Acute and steep rise of ICP after infusion of minimal volume, signifying severe disturbance of CSF circulation, with ICP rising > 40 mmHg despite seemingly “normal” baseline pressure. (**D**): normal/borderline CSF dynamics, clinical monitoring might be the best choice until clearer disturbances arise. (Figure modified from M.Czosnyka, 30 Lectures on Brain Physics, https://www.neurosurg.cam.ac.uk/research-groups/brain-physics-lab/brain-physics-lectures-2020/?fbclid=IwAR0A6Yh-NqAheMO7GHi2HHD-Bug5mgSyhK7nWM1uG729wbt4a6XNqOzFZQ4, accessed on 13 April 2021).

Ultimately, without assessing outcome, the CSF dynamics perspective suggests the following; see Figure 13.

**Figure 14:**
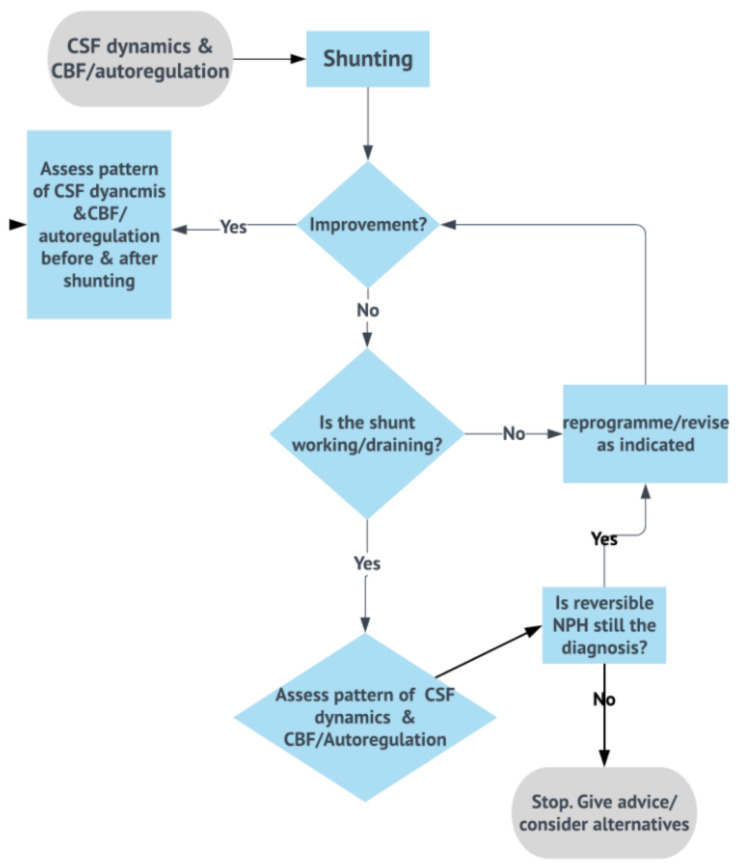
Summary of CSF dynamics testing applied to the bedside. This is a summary of lessons learned from testing CSF dynamics (on its own or combined with cerebrovascular markers) in shunted patients. Main methodological and physiological applications involve understanding and testing cerebral circulation using objective and feasible methods. It also requires understanding that shunt implantation involves understanding how shunt’s function influences success of intervention. This algorithm by no means represents any clinical recommendation, as there are currently no trials to validate our highlighted scientific principles.

To improve the utility of the CSF infusion test in relationship to clinical outcome and based on the overview given at the current review, the shunt process can be the following (combined CSF dynamics, CBF, and shunt testing in vivo approach)—Figure 14.

Again, please be reminded that this is not a clinical or guidance proposal, it is simply the logical derivative of the perspective and overview we have provided in this work.

## 4. Conclusions

Rout is an important biological marker of CSF circulation. It should not, however, be used as a single predictor for improvement after shunting. No single parameter from the CSF infusion test could undertake this role either. Testing of CSF dynamics provides a bedside tool to enhance our understanding of NPH and the process of its reversal. It can be combined with other tools, support clinical practice, and become a strong ally to neurosurgeons who aspire to practice precision medicine. It allows to diagnose the hydrodynamic properties of the cerebrospinal compartment, the system, which is being modified by a shunt, hence altering its hydrodynamic properties. Therefore, it provides the baseline for patient’s management. We emphasize the importance of shunt testing in vivo both in the context of suspecting malfunction, as well as in improving management of the patient and outcome assessment in NPH. Our experience of nearly 30 years of studying CSF dynamics in patients requiring shunting and/or shunt revision, combined with all the recent progress made in producing evidence on the clinical utility of CSF dynamics, has led to reconsidering the discrepancies between CSF circulation testing and clinical improvement.

Patients have changed in the last 50 years since the early works on Rout and infusion tests; neurosurgery has changed and shunt technology is moving forward. We could attempt at adapting infusion tests and our objective investigations, the only stable constant of the last 4–5 decades, to keep up with those changes and find out whether they could serve this purpose or not. Further studies on this direction could help us address this issue and reach a consensus.

## Figures and Tables

**Table 1:** Studies of Resistance to CSF outflow (Rout) and outcome after shunting in NPH: prediction of outcome after shunting. LR: Likelihood ratio, PPV: positive predictive value, NPV: negative predictive value. Mixed Aetiology refers to NPH of all causes.

Reference	NPH	Aetiology	Rout	PPV	NPV	Other Main Findings
Borgensen et al. (1982) [104]	80	Mixed	≥12	96–100%	>95%	NA
Borgensen et al. (1989) [9]	183	Mixed	≥12	NA	100%	NA
Boon et al. (1997) [10]	101	iNPH	≥12 & ≥18	80% & 92%	34%	Highest LR of 3.5 for Rout 18
Kahlon et al. (2002) [47]	68	iNPH	≥14	80%	NA	Strong correlation between Rout & Outcome
Wikkelso et al. (2013) [11]	115	iNPH	≥12 & ≥18	86 & 94%	18 & 18%	No correlation of Rout & Outcome
Nabbanja et al. (2016) [105]	310	Mixed	≥13 & ≥18	NA	NA	Rout correlated with outcome. Kruskall-Wallis Value >6.5 and >6.
Lalou et al. (2020) [106]	369	Mixed	≥13 & ≥18	NA	NA	Rout > 18 highest Chi-square: 9.7 if age-adjusted

NA—Not available.

## Data Availability

Not applicable.
